# The Revisited Genome of *Bacillus subtilis* Bacteriophage SPP1

**DOI:** 10.3390/v10120705

**Published:** 2018-12-11

**Authors:** Lia M. Godinho, Mehdi El Sadek Fadel, Céline Monniot, Lina Jakutyte, Isabelle Auzat, Audrey Labarde, Karima Djacem, Leonor Oliveira, Rut Carballido-Lopez, Silvia Ayora, Paulo Tavares

**Affiliations:** 1 Institut de Biologie Intégrative de la Cellule (I2BC), French Alternative Energies and Atomic Energy Commission (CEA), Centre National de la Recherche Scientifique (CNRS), Univ Paris-Sud, Université Paris-Saclay, 91190 Gif-sur-Yvette, France; lia.marques-godinho@i2bc.paris-saclay.fr (L.M.G.); mehdi.elsadekfadel@i2bc.paris-saclay.fr (M.E.S.F.); Isabelle.AUZAT@i2bc.paris-saclay.fr (I.A.); audrey.labarde@i2bc.paris-saclay.fr (A.L.); karima.djacem@gmail.com (K.D.); Leonor.OLIVEIRA@i2bc.paris-saclay.fr (L.O.); 2 MICALIS, Institut National de la Recherche Agronomique (INRA), AgroParisTech, Université Paris-Saclay, 78350 Jouy-en-Josas, France; celine.monniot@jouy.inra.fr (C.M.); rut.carballido-lopez@inra.fr (R.C.-L.); 3 Unité de Virologie Moléculaire et Structurale (VMS), CNRS, 91198 Gif-sur-Yvette, France; lina.jakutyte@gmail.com; 4 Centro Nacional de Biotecnología (CNB-CSIC), 28049 Madrid, Spain; sayora@cnb.csic.es

**Keywords:** Bacteriophage, *Siphoviridae*, SPP1, *Bacillus subtilis*, genome organization, DNA replication, virus assembly, virus DNA packaging, virus evolution

## Abstract

*Bacillus subtilis* bacteriophage SPP1 is a lytic siphovirus first described 50 years ago. Its complete DNA sequence was reported in 1997. Here we present an updated annotation of the 44,016 bp SPP1 genome and its correlation to different steps of the viral multiplication process. Five early polycistronic transcriptional units encode phage DNA replication proteins and lysis functions together with less characterized, mostly non-essential, functions. Late transcription drives synthesis of proteins necessary for SPP1 viral particles assembly and for cell lysis, together with a short set of proteins of unknown function. The extensive genetic, biochemical and structural biology studies on the molecular mechanisms of SPP1 DNA replication and phage particle assembly rendered it a model system for tailed phages research. We propose SPP1 as the reference species for a new SPP1-like viruses genus of the *Siphoviridae* family.

## 1. Introduction

The lytic bacteriophage SPP1 (Subtilis Phage Pavia 1) that infects the soil bacterium *Bacillus subtilis* was isolated in the Botanical Garden of Pavia in Italy [1]. The capacity of its purified DNA to transfect *B. subtilis* competent cells and the difference of density between its two DNA strands, rendering easy their separation following denaturation, were quickly recognized. They attracted initial interest to this phage system for studying DNA uptake into bacteria [2,3], mismatch repair [2,3] and recombination [4,5,6]. Establishment of the genetic [7] and physical [8,9] maps of the SPP1 genome then paved the way for studies of phage gene expression, DNA replication and assembly of the viral particle. The intensive research that followed on the molecular mechanisms supporting SPP1 infection rendered it one of the best characterized phages of Gram-positive bacteria. SPP1 generalized transduction was also instrumental for fine genetic mapping of the *B. subtilis* chromosome, strain construction, and plasmid horizontal transfer [5,10,11,12,13,14].

SPP1 belongs to the family *Siphoviridae*. Its viral particle has an isometric icosahedral capsid with a diameter of 61 nm [15] and a 190 nm-long non-contractile tail [16]. The linear double-stranded DNA (dsDNA) molecule contained in the viral capsid has a length of ~45.9 kbp with a variation in size of ~2.5 kbp [17,18]. The phage genome size is 44,016 bp. The DNA molecules in a phage population are terminally redundant and partially circularly permuted, resulting from a headful packaging mechanism [19,20].

SPP1 infection is initiated by reversible adsorption of the viral particle to glycosylated teichoic acids [21] followed by irreversible binding of SPP1 to YueB. This integral membrane protein is a component of a type VII secretion system [22] that crosses the *B. subtilis* cell wall to be exposed at the bacterial surface [23,24,25]. The interaction of the SPP1 tail fiber with YueB triggers ejection of SPP1 DNA through its tail tube, committing the phage to infection. Phage DNA circularizes in the bacterial cytoplasm most probably by recombination between its redundant ends [26,27,28,29,30]. DNA replication then ensues in a discrete position of the bacterial cytoplasm [24]. DNA synthesis was proposed to initiate by theta replication of circular molecules followed by a switch to rolling circle (sigma) replication that generates concatemers of the SPP1 genome [30,31]. These are the substrate for phage DNA encapsidation into a preformed procapsid structure. Packaging is initiated by specific recognition and cleavage of a *pac* sequence within the SPP1 genome, followed by its translocation into the procapsid interior through a specialized portal vertex. Encapsidation is terminated by an imprecise [18], sequence-independent, endonucleolytic cleavage of the substrate concatemer when a threshold amount of DNA is reached inside the capsid (headful packaging mechanism) [19,20]. Subsequent encapsidation cycles follow processively along the concatemer. After packaging, DNA is retained inside the capsid by proteins that close the portal vertex and build the interface for attachment of the phage tail. Binding of the tail, which is assembled in an independent pathway, yields the infectious particle (virion). The SPP1 tail is formed by the adsorption apparatus, responsible for interaction with the host cell surface, which is connected by a 160-nm long helical tail tube to the tail tapered end that binds to the capsid portal [16,32]. Virions accumulate in the infected bacterium until lysis. Lysis is promoted by membrane proteins (holins) that concentrate in the cytoplasmic membrane leading to its disruption and release of an endolysin that digests the *B. subtilis* cell wall [33].

Here we revisit the sequence and organization of the bacteriophage SPP1 genome. A detailed updated annotation of its genes combining available experimental data and bioinformatics completes this comprehensive study of SPP1 genetics and biology.

## 2. Materials and Methods

The SPP1 genome was fully sequenced using Illumina sequencing of genome libraries at the I2BC NGS platform facility, as described [34,35]. Sanger sequencing of PCR fragments covering the SPP1 genome was carried out at GATC Biotech (Germany).

Open reading frames (ORFs) identification and visualization of the SPP1 chromosome organization was performed using the SnapGene Viewer software (GSL Biotech, Chicago, IL) and Fgenesb annotator (http://www.softberry.com/berry.phtml?topic=fgenesb&group=programs&subgroup=gfindb) [36]. A cut-off of ORFs initiated at AUG, GUG or UUG codons that code for putative proteins of at least 45 amino acids-long was used as criterion to identify the ORFs for annotation. Among those, there are 20 ORFs without ribosome binding site (RBS) that are embedded in longer ORFs. Such internal ORFs were eliminated from our downstream analysis because they are most likely not translated. The strong distribution bias of ORFs coded by the SPP1 DNA heavy strand together with transcriptional and functional data (compiled in [26]) support that this strand is used, possibly exclusively, as the coding DNA template. Therefore, the analysis described here is limited to 80 ORFs, defined according to the above criteria, which are encoded by the heavy strand. The assignments made in [26] and a survey of SPP1 functional studies were used as the primary information for ORF annotation. Visual inspection of the ORF 5′ region was used to confirm the initiation codon and to assess if it is preceded by an RBS whose sequence is complementary to the *B. subtilis* 16 S rRNA 3′ sequence UCUUUCCUCCACUAG [37]. A spacing of 8–14 nucleotides was allowed between the center of the RBS (complementary to the U underlined) and the nucleotide preceding the initiation codon. Sequence and structural homology searches of the ORFs encoded proteins were carried out with BLASTp [38,39] and HHPred [40], respectively. Protein properties were analyzed with the Expasy ProtParam tool (https://web.expasy.org/protparam/). Prediction of transmembrane helices was carried out with the TMHMM server v.2.0 (http://www.cbs.dtu.dk/services/TMHMM/).

SPP1 putative early promoters were predicted using BPROM. BPROM recognizes promoter sequences bound by bacterial sigma factors of the σ^70^ family with about 80% accuracy and specificity (http://www.softberry.com/berry.phtml?topic=bprom&group=programs&subgroup=gfindb) [36]. These include promoters recognized by the primary sigma factor σ^A^ of *B. subtilis*. Promoter sequence prediction was carried out within the regions where early promoters were previously mapped [26,41,42]. The −10 and −35 sequences of late promoter *P*L1 were used for word scanning of the SPP1 genome to search for putative late promoters without success. Rho-independent transcriptional terminators were determined using ARNold (http://rna.igmors.u-psud.fr/toolbox/arnold/index.php) [43,44,45,46], performing a whole sequence search analysis on the coding strand using two complementary programs, Erpin and RNAmotif. The free energy (ΔG°) of the predicted terminator stem-loop structure was computed with ARNold [44].

Codon usage bias of the 80 SPP1 ORFs was calculated using the Codon Usage Calculator from Biologics International Corp (https://www.biologicscorp.com/tools/CodonUsageCalculator/). The *B. subtilis* genome codon usage frequencies were obtained from [47,48].

The revised version of the complete SPP1 genome sequence and its annotation are in the process of submission to Genbank with accession code “X97918.3”. Table 1 summarizes the changes identified relative to the previous sequence.

## 3. Results and Discussion

### 3.1. Properties of the SPP1 DNA Molecule

**Figure 1:**
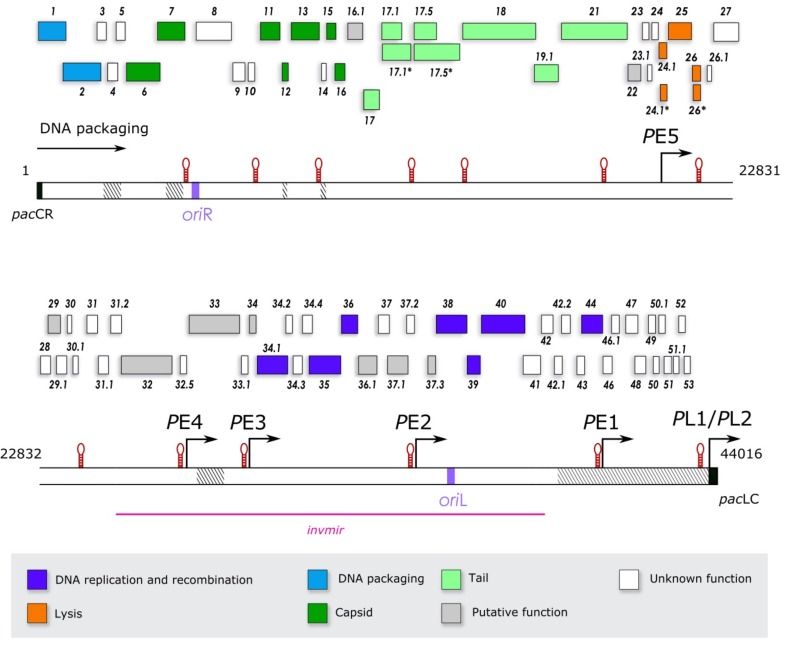
Organization of the SPP1 genome. The continuous bar represents the 44,016 bp-long genome where coordinate 1 is the main *pac* cleavage position [35]. The two origins of replication *ori*R and *ori*L (magenta), the DNA packaging signal *pac* (black) and non-essential regions of the SPP1 genome defined by deletions (dashed) are highlighted in the bar. The sequence inverted in SPP1*invmir* is displayed by a pink line underneath the genome bar. The position of promoters (Figure 2) and potential Rho-independent transcriptional terminators that form stem loops (red) in mRNA (Table 2) is displayed on top of the bar. Transcription is from left to right. DNA packaging initiated at *pac* occurs in the same direction (arrow on the top left). The set of SPP1 genes and ORFs, identified as described in Materials and Methods (see Section 2), are presented above the genome bar and colored according to their function assignment shown on the bottom legend.

The genome of SPP1 was completely sequenced in the context of research projects on phage DNA replication and viral particle assembly, as well as by the necessity for an accurate genome annotation for downstream SPP1 “omics” studies. Very few changes were identified relative to the deposited sequence (Genbank accession code X97918) confirming the high quality of the original Sanger sequencing work [26] and its 2006 revision (X97918.2; [16]). Changes in the nucleotide sequence are listed in Table 1. The 44,016 bp genome has a GC content of 43.7% which is similar to the one found in the host *B. subtilis* genome (43.5%) [49]. The distribution of purines and pyrimidines in the two DNA strands is very different. This asymmetry provided the physical basis for their separation after denaturation by density using isopycnic centrifugation [1]. The heavy strand, whose purine (dA+dG) content is 58.4%, has a density (ρ) of 1.725 g/cm^3^. It is the SPP1 genome coding strand. The light chain density is 1.713 g/cm^3^ [1,3]. The strongest sequence bias are dA+dT rich-islands found in intergenic regions involved in transcriptional regulation (see Section 3.2) and in the two origins of SPP1 DNA replication *ori*R and *ori*L (see Section 3.5) (Figure 1).

### 3.2. Transcription and Translation of the SPP1 Genome

**Figure 2:**
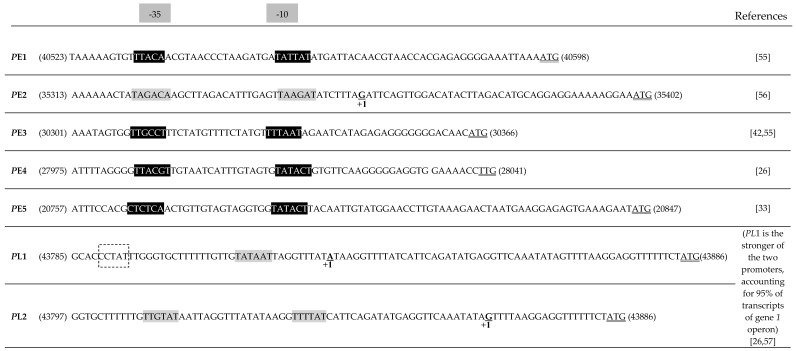
SPP1 promoters. The sequence of SPP1 promoters and the initiation codon of their downstream gene (double underline) are displayed. The −35 and −10 promoter regions are shaded in grey for promoters whose transcription start position (+1) was determined experimentally. A dashed box denotes the atypical −35 sequence of *P*L1. SPP1 putative early promoters identified by sequence similarity to *B. subtilis* vegetative promoters are highlighted in black with sequence characters in white. Note that the approximate position of transcription initiation and promoter strength was determined for all early promoters by electron microscopy of DNA-RNA polymerase complexes [41,42].

Transcription of the SPP1 genome was reported to occur from the DNA heavy strand [1,50,51,52]. This is not an absolute requirement, as phage SPP1*invmir* carries an inversion within its genome that leads to transcription from the light chain of a large genome segment which includes the DNA replication genes (Figure 1) [53]. The temporal gene expression program is defined by early and late transcription carried out by the *B. subtilis* RNA polymerase [54]. Five promoters with a canonical sequence recognized by *B. subtilis* σ^A^ direct transcription of early ORFs/genes (we name gene the ORFs for which a function was demonstrated experimentally or supported by substantial bioinformatics data) (Figure 1 and Figure 2) [33,41,55,56]. These polycistronic transcriptional units are bracketed by the promoter and by putative Rho-independent transcriptional terminators coding for potential RNA stem-loop-forming sequences (Figure 1 and Table 2). The two strongest promoters (*P*E3 and *P*E2) drive transcription of gene sets that include essential DNA replication functions (Figure 1) [41,52,56]. *P*E1, also a strong promoter, and the weaker *P*E4 and *P*E5 promoters control expression of less characterized ORFs (see Section 3.4). Early genes/ORFs are encoded contiguously in the SPP1 genome. Transcription of the late genes segment requires translation of (an) early, yet unidentified, SPP1 factor(s) [55,57]. The only late promoters experimentally characterized are the adjacent *P*L1 and *P*L2 (Figure 1). *P*L1, which accounts for more than 95% of the transcriptional activity of the region upstream from gene *1*, has a canonical −10 sequence and an atypical −35 region ([57]; Figure 2). Several clusters of late genes are delimited by potential stem-loop transcription terminator sequences ([26]; Figure 1), mostly found in intergenic regions, but no consensual late promoter sequence related to *P*L1 was identified.

Genome-wide sequence analysis and subsequent individual inspection identified 80 ORFs encoded by the SPP1 wild type DNA heavy chain with a length longer than 45 codons (135 bp), as described in Material and Methods (see Section 2) (Table 3; Figure 1). A stringent criterion was used to annotate only ORFs that are most likely translated, an assignment further supported by the subsequent search for their 5′ RBS (see below). The ORFs defined cover 94% of the SPP1 sequence. Non-coding segments longer than 45 bp were characterized in most cases by the presence of transcriptional promoters and/or terminators sequences, but were also found in five cases between adjacent genes within a transcriptional unit (genes *24*–*24.1*, *26.1*–*27*, *31*–*31.1*, *46.1*–*47*, *48*–*49*). Only one ORF longer than 15 codons was identified in these intergenic regions. It has 33 codons starting by AUG and preceded by an RBS in the segment between ORFs *46.1* and *47* (not shown). It cannot be excluded at present that other regions of the genome code polypeptides smaller than 45 amino acids-long, as found for several phages [58,59]. We are pursuing proteomic and genetic studies for an accurate annotation of short SPP1 ORFs. An RBS whose sequence is complementary to the *B. subtilis* 16 S rRNA 3′ was found in the 5′ region of 77 from the 80 ORFs investigated here (Table 3). No RBS was identified for genes *2* and *16* that encode well-characterized proteins essential for phage particle assembly (Table 3). Note that in case of gene *16* the initiation codon was confirmed experimentally by amino-terminus sequencing of its encoded protein [60]. The initiation codon of gene *2* overlaps the stop codon of its 5′ gene *1* (**AUG**A; the initiation codon is shown in bold and the termination codon in double underlined) while the initiation codon of gene *16* is spaced by one nucleotide from the gene *15* termination codon (UAAG**AUG**). This organization possibly allows coupling initiation of translation of the genes lacking an RBS with translational termination of their upstream gene [61]. It might also provide a strategy to control the ratio between proteins encoded by adjacent genes. Those proteins interact directly during phage assembly: gene product 1 (gp1 (note that the designations gpX (gene product X) and G*X*P (gene *X* product) are synonymous in the SPP1 literature)) and gp2 form the DNA packaging terminase while gp15 and gp16 bind sequentially to the capsid portal after viral DNA packaging (see Section 3.8). ORF *42.2* also lacks a canonical RBS. Its initiation codon overlaps the termination codon of ORF *42.1* (**AUG**A) and is preceded by a GGGG sequence that could act as a weak RBS (Table 3), probably ensuring gp42.2 synthesis. It is possible that other SPP1 ORFs without RBS, a situation found for ~10% of the host *B. subtilis* ORFs [62], are expressed during SPP1 infection. These can be either smaller than 45 codons or embedded within SPP1 annotated ORFs (see Section 2).

AUG is the translation initiation codon of 72 ORFs (90%) of the SPP1 genome. It is found in all genes coding proteins of known function with the exception of the tail spike gene *21* that starts with UUG (Table 3). ORFs *27* and *32.5* also initiate with UUG while five other ORFs start with GUG (6%). These percentages differ somehow from the ones found for *B. subtilis* ORFs but AUG remains the initiation codon most frequently used (78%) by the host bacterium [110]. A significantly higher frequency of UGA termination codons (39%) is found in the SPP1 genome when compared to *B. subtilis* (24%). This is accounted by a reduction of UAA codon usage from 62% in *B. subtilis* to 50% in SPP1 (Table 4). UAG remains the rarest codon in the two genomes. The overrepresentation of UGA in SPP1 is surprising because there is an abnormally high 6% read-through of this codon in *B. subtilis* that extends the length of polypeptide chains [111]. However, the frequency of UGA is reduced to 29% when considering the SPP1 genes of known function that play key functions in DNA replication, viral particle assembly, and cell lysis (the “core” genome). In case of gene *6*, which encodes the essential capsid portal protein, correct polypeptide chain termination is ensured by an arrangement of stop codons in tandem (UGAUAA). Furthermore, all genes coding proteins required in high amounts for phage particle assembly feature a UAA stop codon (hundreds of copies of the (pro)capsid proteins gp11, gp12, gp13 as well as of the major tail tube protein (TTP (note that TTPs are also designated major tail proteins (MTPs)) gp17.1 are used for assembly of one viral particle (see Section 3.8)). The global codon usage frequency shows also some differences between SPP1 and *B. subtilis* (frequency differences above 10% are highlighted in bold and rare codons (<10% frequency) are underlined in Table 4).

There are two documented cases of programmed translational frameshifts in SPP1. These recoding events result from slippage of ribosomes into a different coding frame during translation of mRNA. The frequency of the frameshift dictates a constant ratio of the two proteins synthesized which have an identical amino terminus but a different carboxyl region sequence. The two TTPs gp17.1 and gp17.1* share an identical sequence, but the gp17.1* carboxyl terminus is extended by 87 additional amino acids [91]. The +1 translational frameshift results of ribosomes pausing at CCC rare proline codons and their shift to the overlapping frequent CCU proline codon in the CCCUAA sequence which codes also for the gene *17.1* termination codon [91]. The amount of gp17.1/gp17.1* synthesized is compatible with their 3:1 ratio found in SPP1 tails. The other programmed frameshift was identified by bioinformatics in genes *17.5* and 17.5* which encode functional analogs of phage lambda gpG/gpGT [91,112]. These are chaperones of tail tube assembly [113,114]. In SPP1, the putative −1 frameshift occurs at a UUUUUUC heptanucleotide slippage sequence within gene *17.5* that leads some ribosomes to change coding frame yielding gp17.5* [91]. Gp17.5* has the first 112 amino acid sequence identical to gp17.5.

Genes with two initiation codons preceded by canonical RBSs for the same coding frame and spaced by a few codons were identified in the two holin genes (genes *24.1*/24.1* and *26*/26*) [33]; see Section 3.6).

### 3.3. Organization and Function of the SPP1 Genes

The 80 ORFs annotated here have a compact arrangement leaving only 6% non-coding sequences in the SPP1 genome. The longest intergenic regions carry transcriptional regulation sequences and, one of them, *ori*R (Figure 1).

The core SPP1 genome is presently composed of 28 genes: (**i**) 13 essential genes for which conditional lethal mutations (suppressor sensitive mutations (*sus*) or temperature sensitive mutations (*ts*)) were obtained by chemical mutagenesis of the overall SPP1 genome [7] (genes *1*, *2*, *6*, *11*, *13*, *15*, *16*, *17*, *17.1*, *35*, *38*, *39*, *40*; see Section 3.5 and Section 3.8); (**ii**) 11 genes coding proteins that are anticipated to act in phage tail assembly (*17.5*, 17.5*, *18*, *19.1*, *21*, *22*; see Section 3.8) or in host cell lysis (*24.1*, 24.1*, *25*, *26* and 26*; see Section 3.6); and (**iii**) 4 non-essential genes whose inactivation is detrimental for SPP1 multiplication (*7*, *34.1*, *36*, and *44*; see Section 3.5 and Section 3.8). Genes *12* and 17.1* are excluded from this group because their inactivation has no detectable effect in SPP1 fitness in laboratory conditions (see Section 3.8). The core genes identified include most of the minimal genetic set necessary for lytic phage multiplication ensuring viral DNA replication, viral particle assembly, and host lysis. A present omission in SPP1 research is dissection of the genetic circuitry that controls genome transcription and, in particular, late genes expression. Several putative DNA-binding proteins encoded by early genes are good candidates to be transcriptional regulators (Table 3; see Section 3.4). Bioinformatics allowed us to assign putative function and/or biochemical activity to genes *16.1*, *29*, *32*, *33*, *34*, *36.1*, *37.1*, and *37.3* (Figure 1; Table 3; see Section 3.4 and Section 3.8). In total, 38 genes were functionally annotated (48% of SPP1 ORFs). They cluster in segments within four early transcriptional units, coding DNA replication and early lysis proteins together with uncharacterized polypeptides, and in one large genome region transcribed late. In this late region the synteny of genes encoding structural proteins of the SPP1 virion and late lysis genes is conserved when compared to the genomes of other *Siphoviridae* (Figure 1).

The function of other SPP1 ORF products is poorly understood. Genome deletions or inversions showed that ORFs *3*–*5*, *7*, *12*, *14*, *31.2*, *32*, *33* and *42*–*53* are non-essential (Figure 1; [26,35,53,55,83,88]; this work; P.T., unpublished) which corresponds to 19% of the SPP1 genome. Among these, a function was assigned experimentally only to the products of genes *7*, *12* (see Section 3.8) and *44* (see Section 3.5) while bioinformatics revealed a putative role for gp32 and gp33 (see Section 3.4). Within the SPP1 genome regions not tested by deletion analysis, the (putative) function of the products from ORFs *8*, *9*, *10*, *23*, *23.1*, *24*, *26.1*, *27*, *28*, *29.1*, *30*, *30.1*, *31*, *31.1*, *32.5*, *33.1*, *34.2*, *34.3*, *34.4*, *37*, *37.2*, and *41* remains unknown. Most of these 22 ORFs are probably non-essential for SPP1 multiplication. In total, 42 ORFs (52% of the total SPP1 ORFs) have no assigned function. Protein sequence (BLASTp) and structural (HHPred) homology searches revealed 18 orphan ORFs among those (*8*, *10*, *14*, *23*, *23.1*, *24*, *28*, *30.1*, *32.5*, *33.1*, *34.3*, *34.4*, *37*, *37.2*, *42.1*, *46.1*, *51.1*, and *52*) whose products are unrelated to protein sequences in databases. The other SPP1 ORF protein products are homologous in their vast majority to proteins with no known function from *Bacillus* spp. or from their phages, suggesting that they belong to a mobile gene pool of bacilli and their viruses. Most of the less characterized SPP1 ORFs are likely additions to the lytic phage core genome resulting from insertions of DNA that add “more on to it” (“morons” [115,116,117]). Morons designated originally complete transcriptional units added to phage genomes [115] but include presently also gene insertions that are unique or found in a limited set of genomes. They frequently encode beneficial features for phage adaptation to its host and environment. We consider SPP1 morons the ORFs *3*–*5*, *8*–*10*, *12*, *14*, *23*–*24*, *26.1*–*28*, *29.1*–*31.2*, *32.5*, *33.1*, *34.2*–*34.4*, *37*, *37.2*, *41*–*43*, and *46*–*53*.

The SPP1 ORFs length is highly variable, encoding proteins with an average length of 179 ± 197 amino acids and an average molecular mass of 20.3 ± 21.9 kDa. The tail tape measure protein and in some cases the tail tip protein, both with more than 1000 residues in SPP1, is (are) the longest protein(s) encoded by phages with long tails. They are landmarks to map the phage tail genes which, when combined with the synteny of genes coding virion assembly proteins, provide a first approximation to the genomic organization of late genes. The SPP1 core genes (most coding proteins >100 amino acids-long) tend to be longer than moron genes (most coding proteins <100 amino acids-long).

A BLASTn of the complete SPP1 genome showed extensive nucleotide sequence homology only to phages of the SPP1 group rho15, SF6, 41c [9,118], and the recently identified Lurz phage series [35]; P.T. unpublished) defining the genetic basis for a SPP1-like *genus*. Strong DNA sequence homology hits to other phages and to *Bacillus* spp. genomes (probably to prophages, defective phages or other elements of the bacilli mobile genome) were limited to a few individual genes (*5*, *9*, *10*, 17.1*, *25*, *33*, *48*, *49*, *50.1*, and *51*). An interesting case is *B. subtilis* (natto) phage PM1 [119] that has four blocks of DNA homology to four SPP1 ORFs/genes (*9*, *25*, *33* and *51*) which are separated by unrelated sequences. Multiple horizontal gene transfer events thus occur within the genetic mobile landscape of *Bacillus* spp. and its phages.

DNA nucleotide homology provides a sensitive criterion to detect recent genetic exchanges, while protein amino acid homology, found for a much larger set of SPP1 gene products, assesses far-reaching functional relationships with proteins from other phages. This is particularly well illustrated by bacteriophage GBK2 that infects the termophilic bacterium *Geobacillus kaustophilus*. Its genome shows no nucleotide sequence homology to SPP1 which infects a mesophilic bacillus. However, a large region encodes proteins homologous to SPP1 gp26 (holin), gp29, gp31, gp31.1, gp32, known DNA replication proteins (gp34.1, gp35, gp36, gp39, gp40), gp42, gp42.2 and gp43 [120] (see Section 3.4 and Section 3.5). Their genes order is conserved in the two phage genomes, being spaced by genes of unrelated proteins. In contrast, phage particle assembly proteins of the two phages lack significant homology, apart from SPP1 proteins gp16.1 and gp17 which have GBK2 homologs, while the ensemble of their coding genes conserves the synteny found in siphoviruses [120] (this study). Thus, the genome of both phages is assembled from a common ancient genome module of early genes, traceable by protein homology, and of an evolutionarily distinct module coding the viral particle assembly proteins. The first module diversified by acquisition and eventual loss of different morons (single genes and also transcriptional units like the one controlled by *P*E4 in SPP1; Figure 1) while the viral particle assembly module acquired the genes *16.1*–*17* cluster, most probably from horizontal exchange into a conserved position of the module. The genomes of SPP1 and GBK2 are a compelling case of tailed phages evolution by combination of modules, horizontal gene transfer, and diversification by morons acquisition/loss [115,116,117,121,122].

In the following Sections we describe current knowledge on SPP1 genes and ORFs with particular attention to those that are less characterized. In-depth reviews on SPP1 biology [123], DNA replication [30], and viral particle assembly [124,125,126] are available.

### 3.4. The SPP1 Genes Set. I. Uncharacterized Early Genes

Putative moron genes are spread throughout the five early SPP1 transcriptional units (Figure 1).

The dispensable set of short ORFs *46* to *53,* under the control of *P*E1, has no known or putative function deduced from bioinformatics. ORFs *46.1*, *53*, and the sequential ORFs *50.1*, *51*, *51.1* have predicted transmembrane segments (Table 3) indicating that these polypeptides insert most likely in the cytoplasmic membrane to achieve early roles in the host cell. Segments of DNA homologous to different combinations of ORFs *48* through *51* are found in different SPP1 phages or *Bacillus* spp. strains, suggesting common functions.

Protein sequence analysis and predicted structural homology led to functional assignments to several genes in transcriptional units controlled by promoters *P*E4 and *P*E5. *P*E4 drives expression of ORFs *32.5* and *33.1*, whose function is unknown, together with the dispensable gene *33*. Gp33 is a 589-long protein that shares 88% amino acid sequence identity with a protein of *B. subtilis* (natto) phage PM1. It is also highly homologous to a large number of proteins from *Bacillus* spp. annotated to have cell wall hydrolysis activity or as proteins of *Bacillus* phages with a predicted right-handed parallel β-helix repeat fold. HHPred extends this structural homology to adhesins binding to the cell wall surface as well as to phage tail spikes of *B. subtilis* phage phi29 and of enterobacteria phages like sf6, HK620, and LKA1. Gp33 could be the trace of a tail fiber used by an ancestor of SPP1, like the Ur-lambda tail fibers lost during laboratory evolution [127]. However, this does not explain why SPP1 maintained gene *33* expressed early during infection in an apparently functional form. We privilege the hypothesis that gp33 accumulates in the cytoplasm and its release upon lysis acts on the wall of the infected bacterium to facilitate lysis and/or on the envelope of other *B. subtilis* cells to facilitate their subsequent infection.

*P*E5 controls expression of 14 ORFs including genes *25*, *26* and 26* involved in cell lysis (see Section 3.6). Most of the other ORFs encode short polypeptides of unknown function. Gp29 and its homologous hypothetical protein of *Geobacillus* phage GBK2 give strong hits in HHPred to DNA-binding proteins with a winged helix fold like transcriptional regulators and excionases. ORFs *30*, *31.1*, and *31.2* products have predicted transmembrane segments suggesting an early role in the cytoplasmic membrane during infection. Gp31.1 and gp31.2, which are encoded by contiguous ORFs, are homologous to numerous proteins of *Bacillus* spp. and their phages, likely sharing a widespread function. The non-essential gp32 (Table 3) is a basic 836-long protein with 75% amino acid sequence identity to a protein of unknown function from *Geobacillus* phage GBK2. Gp32 has also extensive similarity to ATP-binding proteins from *Bacillus* spp. and its phages. HHPred reveals structural homology of its carboxyl terminus, with high confidence scores, to the conjugation protein TrwB, to VirB4 of type IV secretion systems and to proteins of the FtsK family. These machines translocate DNA or proteins across the bacterial membrane. Since gp32 has no predicted transmembrane segments, it could act together with gp31.1 and/or gp31.2 to build a trans-membrane translocon of macromolecules. The synteny of genes coding for proteins homologous to gp31, gp31.1 and gp32 is found also in phage GBK2 [120] suggesting a conserved activity. The exact protein composition, functionality and role of such potential machine remain to be established.

The strongest early promoters, *P*E3 and *P*E2 [41], drive expression of DNA replication genes (see 3.5) that alternate with genes coding uncharacterized proteins (Figure 1). Bioinformatics allowed us to assign putative activities to four proteins of the latter group. Gp34 is a putative 6.3 kDa basic polypeptide with predicted structural homology to DNA binding proteins, whose strong hits are transcriptional repressors. Its small size advises, nevertheless, some caution on this functional assignment. Gp36.1 is highly homologous to HNH endonucleases of Gram-positive bacteria, and HHPred uncovered a relationship to the structure of *B. subtilis* phage SPO1 HNH homing endonuclease I-HmuI (PDB accession number 1U3E) [128]. Proteins containing the HNH motif carry out intron homing in phages T4, SPO1 and SP82 [129,130], and are essential for DNA packaging in a group of *cos*-phages [131,132]. In other phages they play dispensable functions like in *Staphylococcus aureus* phage 80α [133]. Gp37.3 is a small basic polypeptide whose amino acid sequence and structure prediction show relatedness to numerous bacterial and phage DNA binding proteins. SPP1 gp34, gp36.1, gp37.3 together with gp29, and possibly other early polypeptides that did not deliver robust bioinformatics hits yet, are strong candidates to participate in SPP1 DNA metabolism and in gene expression regulation processes calling for further research. Gene *37.1* that is found within this set of genes codes for an enzyme with a distinct function. Gp37.1 is highly homologous to poly-γ-glutamate (γ-PGA) hydrolases from phages of Gram-positive bacteria, mainly infecting *Bacillus* spp. like vB BsuM-Goe3, BSP10, Grass, BSNPO1, PM1, phiNIT1, PBS1, AR9 and others. HHPred provided a strong hit to the structure of the γ-PGA hydrolase PghP from phage phiNIT1 (PDB accession number 3A9L). The supposed role of the enzyme in phages is to degrade the γ-PGA polymer layer. This layer forms a shield at the surface of several microorganisms, mainly *Bacillus* spp., to protect them from environmental attacks as diverse as phagocytosis or phage infection [134,135]. Release of γ-PGA hydrolase from phage-infected bacteria would thus open the way for infection of new host bacteria protected by a γ-PGA layer. Experimental demonstration of γ-PGA hydrolase activity from *B. subtilis* phages phiNIT1 (protein PghP; [136]), BSP10 [137], as well as from prophages SPβ and prophage-like element 5 (YokZ and YndL, respectively) [135] support this hypothesis.

The large number of early ORFs not conserved among SPP1 and other tailed phages, whose majority has an unknown function, is a rich and diverse patrimony. We anticipate that this genetic pool is mostly dedicated to strategies engaged at the beginning of infection to take over the host cell [138] (and references therein), possibly to mediate superinfection exclusion of the infected bacterium by other phages [139,140] (and references therein), and/or to accumulate molecules that will be released upon lysis to support subsequent infection of new host bacteria [134,135,136,137]. Most of the phage effectors involved are dispensable (e.g., the proteins encoded by transcriptional units under the control of *P*E1 and *P*E4 in SPP1) but their combination might provide a determinant fitness advantage, globally or in specific environmental settings, to SPP1-like phages.

### 3.5. The SPP1 Genes Set. II. DNA Replication Early Genes

The bacteriophage SPP1 DNA replication is a well-characterized process. SPP1 was reported to have two origins of replication, *ori*R and *ori*L (Figure 1), localized ~13 kbp apart in the circularized genome. The two sequences have a similar organization composed of direct repeats and an AT-rich region that acts as a DNA unwinding element (DUE) [102]. The SPP1 origin binding protein gp38 binds to the direct repeats of *ori*L, positioned within gene *38* (Figure 1), and of *ori*R with nanomolar affinity [102]. Replication initiation requires gp38, the helicase loader gp39 [103,104] (PDB accession number 1NO1), and the replicative helicase gp40 that belongs to the DnaB family [103,105,106] (PDB accession number 3BGW). After melting of the origin sequence and unwinding, the gp40 hub interacts with the host DnaG [107,108] and with the DnaX subunit of the clamp loader [109]. These interactions recruit the host replisome. The theta replication reaction was reconstituted in vitro with a supercoiled plasmid bearing *ori*L and purified SPP1 gp38, gp39 and gp40 together with *B. subtilis* DNA polymerases PolC and DnaE, the processivity clamp DnaN, the hetero-pentameric clamp loader complex (3xDnaX+YqeN+HolB), the primase DnaG, DNA gyrase, and a single-stranded DNA binding protein (SSB) (bacterial SsbA, or SPP1 gp36) [141]. Cellular SsbA can replace the non-essential SPP1-encoded SSB gp36 in SPP1 DNA replication. In contrast, gp36 does not support *B. subtilis* DNA replication and acts in vitro as an inhibitor of the cellular process [101].

After a few rounds of circular molecules DNA synthesis, SPP1 DNA replication switches to generate linear head-to-tail concatemers [28,29] which are the substrate for DNA encapsidation into viral particles. The switch was proposed to be triggered by stalling of the replication fork, probably when it collides with gp38 firmly bound to *ori*R [30,142]. Four SPP1 proteins are likely involved in the process of resuming DNA replication after stalling: the Holliday junction resolvase gp44 [31], the 5′→3′ exonuclease gp34.1 [99], the ATP-independent single-strand annealing recombinase gp35 [100], and the SSB gp36 [101]. The current model is that they would act coordinately to process the stalled replication fork and make a double-strand break, followed by homology-directed recombination generating the template for DNA replication by a rolling circle mode (sigma-type) [30,31,142]. A DNA substrate assembled in vitro which mimics the sigma template was used to show that subsequent rolling circle replication is achieved by the same set of proteins that carry out theta-type DNA replication, with the exception of DNA gyrase that is not necessary [101]. The assays also highlighted that gp38 may act like the bacterial PriA protein, because no specific DNA region (*ori*) is needed for this reaction and DNA replication can start at any site after fork pausing [101]. Gp35 recombinase is the only SPP1 essential protein among the switch putative effectors. The lack of gp34.1 and gp36 is detrimental while deletion of the gp44-encoding gene has only a marginal effect in SPP1 viability [13]. Functionally related cellular proteins most likely (partially) compensate for the roles of those viral effectors. Homologs of the SPP1 DNA replication proteins are found in many phages, suggesting that such recombination-driven strategy is a common mechanism to produce concatemers.

The SPP1 replication proteins are encoded by two transcriptional units under the control of promoters *P*E2 and *P*E3 (Figure 1), as in phage GBK2 that shares a similar operon organization and gene synteny [120]. Genes *38*, *39*, and *40* whose products initiate DNA replication form a cluster, but the other DNA replication genes are spaced by ORFs coding proteins with putative DNA binding, host takeover, or unknown functions (see Section 3.4). The selective pressure to cluster genes encoding proteins that closely interact [143] appears stronger for the viral particle assembly genes module (see Section 3.8) than for the DNA replication genes module.

### 3.6. The SPP1 Genes Set. III. Lysis Early and Late Genes

Tailed phage lysis cassettes code typically for a holin that inserts in the cytoplasmic membrane to create holes and for an endolysin that hydrolyses the cell wall leading to efficient bacterial disruption [33,98,144]. Interestingly, The SPP1 genome codes two holin proteins that localize in the cytoplasmic membrane (gp24.1 and gp26) and an endolysin (gp25 or LysSPP1). They act together to lyse *B. subtilis* at the end of the infectious cycle allowing viral particles to escape from the host [33]. Strong nucleotide homology was only detected between gene *25* and the endolysin gene of phage PM1 [119] but proteins homologous to gp24.1, gp25 and gp26 are encoded by numerous phages of Gram-positive bacteria.

A lysis system with two holins (XhlA and XhlB) combined with an endolysin (XlyA) was also described for the defective *B. subtilis* phage PBSX [145]. XhlA and XhlB are homologous to SPP1 gp24.1 and gp26, respectively. In SPP1 their coding genes flank the endolysin gene *25*, while the two holin genes precede the endolysin gene in the PBSX genome. Gp24.1 and XhlA feature a predicted transmembrane helix in their carboxyl terminus while gp26 and XhlB have the canonical organization of phage holins with two transmembrane segments. Each individual holin of these phages does not appear detrimental to *B. subtilis*, while their co-production leads to immediate cell death followed by lysis, showing that the two holins cooperate for disruption of the bacterial membrane [33,145].

Both SPP1 genes *24.1* and *26* have one in-frame internal initiation codon AUG preceded by a correctly spaced RBS (Table 3). Translation started at these AUG codes gp24.1* and gp26* which lack the first 9 and 2 amino terminus amino acids when compared to gp24.1 and gp26, respectively. Such dual start motifs leading to synthesis of two highly related proteins might be involved in lysis regulation as found for the phage lambda S protein whose longer polypeptide has an antiholin function that counteracts the shorter polypeptide holin activity [146]. In contrast to most phages, SPP1 genes *25* and *26* are transcribed early during infection from promoter *P*E5 localized within the gene *24.1* coding sequence [33]. It is likely that the production of gp24.1, which results from late transcription, defines the tempo for the three SPP1 lysis proteins to concur for disruption of the infected bacterium.

### 3.7. The SPP1 Genes Set. IV. Uncharacterized Late Genes

The SPP1 late genome region has an order of genes coding proteins involved in assembly of the viral particle that is conserved among numerous tailed phages. This arrangement is interrupted by a genome segment, bracketed between transcriptional signals, which codes for the origin of replication *ori*R and three moron genes (Figure 1). Gp8 and gp10 are unrelated to known proteins, while gp9 is highly homologous to a protein from *B. subtilis* (natto) phage PM1 [119] and to numerous hypothetical proteins encoded by *Bacillus* spp. The function of these SPP1 proteins and if they are necessary for phage amplification remain to be established. 

The genome segment between the tail fiber gene *21* and the lysis proteins encodes also four proteins whose precise role is unknown. Gp22 has homology to a group of *Bacillus* spp. putative proteins and its structure (PDB access code 2XC8) reveals a fold similar to a domain of the tail receptor binding protein from lactococcal phage p2 [96] (see Section 3.8). The structure of the gp23.1 hexamer was also determined (PDB access code 2XF7) but provided no conclusive insight on protein function [97]. Gp23, gp23.1 and gp24 gave no strong homology hits to proteins in the databank.

The role of the dispensable short ORFs *3*, *4*, *5* and *14* that separate essential genes for SPP1 capsid assembly is not known. The products of ORFs *3* and *4* show homology to a hypothetical protein of *Bacillus* phages vB_BsuM-Goe3 and PM1, respectively, while gp5 has similarity to a large number of bacterial and phage uncharacterized proteins. ORF *14* is an orphan.

### 3.8. The SPP1 Genes Set. V. Viral Particle Assembly Late Genes

SPP1 devotes ~40% of its genome information to assembly of the SPP1 viral particle, a process that was extensively studied. DNA-filled capsids (nucleocapsids) and tails are built in two independent assembly pathways. The two structures then join in a final reaction to yield the infectious particle (virion), like in all studied phages with a long tail.

Construction of the SPP1 nucleocapsid follows the similar assembly pathway used by viruses of the tailed phages-herpesviruses lineage [147,148] (and references therein). A spherically shaped icosahedral DNA-free procapsid with a diameter of ~55 nm is formed first. It is built by polymerization of the major capsid protein (MCP) gp13 that is chaperoned by the internal scaffolding protein gp11 [85,86,87]. One of the 12 vertexes of the procapsid icosahedron is a specialized structure defined by presence of a cyclical dodecamer of the portal protein gp6 ([20,60,75,76,77,78,79,80,81]; PDB access code 2JES). Assembly of the procapsid initiates most likely at the portal vertex by co-interaction between gp6, gp11 and gp13. In absence of gp6, gp11 and gp13 assemble procapsids of normal and of a smaller size, revealing that the portal ensures correct size determination of the procapsid [86]. Co-production of gp6, gp11 and gp13 is sufficient and necessary for assembly of procapsids functional for DNA packaging, showing that they are the minimal set of essential components to build these structures [86]. Gp6 makes a strong interaction with gp7 that targets this protein to the procapsid interior in a few number of copies [84]. This strategy was likely exploited in numerous lysogenic phages for targeting to their capsid interior a toxin fused to the carboxyl terminus of gp7-like proteins (also designated Mufs) [149]. The toxin was proposed to be subsequently delivered to the host at the beginning of infection [149,150]. SPP1 gp7 is a dispensable component of the virion. Phage assembly is not affected in its absence, but only ~25% of virions lacking gp7 are infectious, showing that it supports initiation of SPP1 infection [83].

Head-to-tail concatemers of the SPP1 genome synthesized during phage DNA replication (see Section 3.5) are the substrate for encapsidation into procapsids. The *pac* sequence in the SPP1 genome [151] (Figure 1) is specifically recognized and cleaved by the SPP1 terminase complex to initiate DNA packaging [70,152]. The terminase is composed of the small subunit (TerS) gp1 that binds to *pac* [57,63,64,65,66] (PDB access code 3ZQQ of gp1 from the SPP1-related phage SF6) and of the large subunit (TerL) gp2, a two-domain protein with ATPase and nuclease activities [57,67,70,71,72] (PDB access codes 2WBN and 2WC9 of the gp2 nuclease domain). Re-analysis of gene *1* sequence showed that gp1 is shorter (Table 1) and more similar to the TerS from other SPP1-related phages [118] than initially reported. The *pac* sequence overlaps the *P*L1/*P*L2 promoters (see Section 3.2), the RBS, and the sequence of gene *1* encoding the gp1 DNA-binding domain (Figure 1) [63]. Gp1 binds to the *pac*L and *pac*R regions of *pac* [63,68] and recruits gp2 for making a double-strand cleavage within *pac*C, a sequence that is flanked by *pac*L and *pac*R [70]. Gp2 has a non-specific nuclease activity [57,67,70,71,72] that is controlled by its appropriate positioning in the gp1-gp2-DNA complex [35]. Cleavage at *pac* is auto-regulated [69] leaving most *pac* sequences uncut in the concatemer used for packaging. This allows encapsidation of DNA molecules longer than the unit-genome length during processive headful packaging (see Section 1) [17,19]. The terminal redundancy of SPP1 encapsidated DNA molecules is essential for their re-circularization at the beginning of infection. The auto-regulated cleavage of *pac* was reproduced in a plasmid minimal system bearing genes *1*, *2* and *pac*, showing that gp1 and gp2 are necessary and sufficient to carry out this reaction in *B. subtilis* [72].

The gp1-gp2-SPP1 DNA complex docks at the procapsid portal vertex to assemble the DNA packaging motor. DNA translocation powered by the ATPase activity of gp2 involves an intricate cross-talk between gp1, gp2, and gp6 [67,73,74,82]. The scaffolding protein gp11 leaves the capsid interior and the capsid lattice undergoes a major conformational change leading to its expansion, maximizing the space for phage DNA packing [85,86]. This process uncovers binding sites at the capsid surface for attachment of gp12 trimers that have a central collagen-like fold [88]. The DNA packaging reaction was characterized in vitro, either using extracts of infected cells [153] or purified linear DNA, gp1, gp2 and procapsids [154]. When a threshold amount of DNA is packaged inside the capsid, the portal protein senses the level of DNA headfilling and triggers the sequence-independent headful cleavage of the DNA concatemer that is most likely achieved by the gp2 nuclease domain [20,77,125,155]. Disassembly of the DNA packaging motor is coordinated with sequential binding of the head completion protein gp15 (PDB access code 2KBZ) to gp6, extending the portal channel, and of gp16 (PDB access code 2KCA) that closes the channel, preventing release of the packaged DNA [60,78,89]. The gp6-gp15-gp16 complex is named connector.

The SPP1 long non-contractile tail has a building plan similar to the long tails of *Siphoviridae* and *Myoviridae*. These structures are structurally related to a variety of cellular, tube-like, delivery devices used in bacterial warfare like phage tail-like bacteriocins (PTLBs), phage tail-like complexes that confer toxicity against eukaryotic cells, or type VI secretion systems [156,157].

The SPP1 tail features a host adsorption apparatus that binds selectively to the *B. subtilis* surface and promotes DNA transfer across the bacterial envelope. This ~31 nm-long structure binds selectively to glycosylated teichoic acids [21], which facilitates its subsequent strong interaction with the membrane protein YueB [23,158], committing the phage particle to infection. The known components of the adsorption apparatus are the tail fiber (or Tal) gp21 [93], which binds YueB [95], and the distal tail protein (Dit) gp19.1 (PDB access code 2X8K) [94]. The gp19.1 hexamer forms a complex with the trimeric gp21 amino terminus that closes the tail tube in the virion and opens at the beginning of infection [93]. Gp19.1 defines one end of the ~160 nm-long tail tube built by a helical array of the TTPs gp17.1/gp17.1* that are found at a ratio of ~3:1 in the tube [91]. Similar tubes can be formed exclusively by gp17.1 in vivo [91] and in vitro [92]. The TTPs form hexameric rings organized around the tape measure protein (TMP) gp18 [16]. The tail tube of sipho and myoviruses is tapered by (a) tail completion protein(s), which provide(s) the interface for tail attachment to the connector. In SPP1 it is the tail-to-head joining protein (THJP) gp17 (PDB access code 2LFP) that interacts with the capsid connector in the viral particle [32,90]. The connector-tail completion proteins structure is named head-to-tail interface [81].

SPP1 tail assembly is anticipated to follow the pathway of phages with long tails typified by T4 [159] and lambda [160]. In such case, the tail adsorption apparatus is formed first providing a platform for initiation of gp17.1/gp17.1* helical polymerization around the TMP gp18. During this reaction gp18 is probably pre-shielded by the chaperones gp17.5 and gp17.5*, as proposed for phage lambda [114]. When the tail tube reaches a defined length, determined by gp18, polymerization stops and tail completion proteins bind to the tube end. Gp17 that is exposed at this end joins the tail to the capsid, a reaction that was characterized in vitro [32].

The function of proteins engaged in assembly of the SPP1 nucleocapsid was defined. They have a large number of homologous proteins from phages or prophages of Gram-positive bacteria that play similar roles in viral particle assembly. The exception is the non-essential capsid accessory protein gp12, whose collagen-like motif is found only in some capsid associated proteins [88] and in tail fibers [161]. The order of genes coding proteins necessary for nucleocapsid assembly follows the conserved genome organization of siphoviruses [162,163,164,165] with the particularity that ORFs *3*–*5*, *8*–*10* and *14* interrupt the usual gene order. The DNA packaging/portal module (coding gp1, gp2, (-, -, -,), gp6, (gp7) spaced by ORFs *8*–*10*, is followed by the icosahedral capsid building module (coding gp11, (gp12), gp13) which is separated by ORF *14* from the head completion proteins module (coding gp15, gp16). Note that in this protein list “-” identifies a protein encoded by a moron gene while proteins that have a known function but are non-essential for SPP1 multiplication are shown within brackets.

Gene *16* is followed by *16.1* and a set of genes whose synteny and protein products are conserved in the tail module of siphoviruses [16,91,162,163,164,165,166,167]. The function of SPP1 gp16.1 and its homologous proteins that are widespread among phages with long tails is yet unknown. Their coding gene is consistently found between the capsid connector and the tail proteins coding genes [166]. The tail module codes for gp17, gp17.1, (gp17.1*), gp17.5, gp17.5*, gp18, gp19.1, and gp21 (Figure 1) [16]. They all have extensive homology to proteins from Gram-positive bacteria and their phages. Gp17.1* and gp17.5*, which result from programmed translational frameshifts (see Section 3.2), feature two domains defined by similarity to different proteins. The amino terminus domains boundaries are roughly delimited by their region of identity with gp17.1 and gp17.5, respectively. The gp17.1* amino terminus shows robust similarity to TTP annotated proteins, while its non-essential carboxyl terminus is homologous to FN3 motifs of proteins from *Bacillus* spp. that are involved in binding to cell surfaces [91]. Their exposure in phage structures suggests a role in adhesion to bacteria [91,168]. The amino and carboxyl domains of gp17.5* have similarity to two distinct sets of proteins with unknown function from Gram-positive bacteria or from their phages. The full-length gp21 Tal has numerous homologs. In addition, a subset of proteins has similarity only to its amino terminus (~400 residues), which is the conserved region of Tal proteins that closes the tail tube [93]. The gp21 long carboxyl terminus interacts with the SPP1 bacterial receptor [95]. This organization is consistent with the modular organization of Tal proteins whose carboxyl termini differs both in length and sequence. Such variation results, namely, from whether they carry or not a domain for interaction with the bacterium and of the selective pressure on this domain to generate diverse strategies to target a specific host [169,170] (and references therein). It remains to be established if proteins gp22, gp23, gp23.1 and/or gp24 (see Section 3.7) which are encoded by genes downstream of gene *21* play a role in tail assembly that is specific for the SPP1 system. Note that the three-dimensional structure of the gp22 monomer shows structural similarity to the shoulder of the tail receptor binding protein of lactococcal phage p2 [96] supporting that gp22 is a SPP1 tail component.

## 4. Conclusions

This reannotation of the SPP1 genome provides an actualized view of our understanding of this phage genetic patrimony and how it supports its multiplication. Phage DNA replication and assembly of the viral particle are particularly well studied, rendering SPP1 one of the forefront systems to understand the molecular basis of these processes in tailed bacteriophages. The available knowledge and tools are excellent assets to pursue research on both themes. Recent work provided also insight on the SPP1 lysis mechanism. The genes involved in these essential steps of the virus cycle occupy ~60% of the SPP1 genome. Much less is known about the function of other genes in spite of 50 years of SPP1 research. One or several of them code yet unidentified proteins that regulate SPP1 gene expression circuitry, a less studied aspect of SPP1 molecular biology. Apart from such regulators, a majority of uncharacterized genes likely code effectors that participate in host cell hijacking to optimize viral multiplication, that provide immunity to super-infection by other phages, or that support phage dissemination in the natural environment. These functions are frequently non-essential for phage survival, having subtle effects that render their study difficult. Transcriptomics, proteomics, and metabolomics combined with systematic knock-outs and sensitive phenotyping assays in different infection settings appears as a promising approach to deliver a complete functional map of SPP1. Those studies will provide insights on the wild side of SPP1 that remains to be explored.

Current knowledge on this phage system and its genetic landscape, that is distinct from other model phages, clearly recommend SPP1 as the reference virus for a new SPP1-like virus genus of the *Siphoviridae* family. The genus includes SPP1-related phages rho15, SF6, 41c [9,118], and the recently identified Lurz phage series [35] (P.T. unpublished) whose DNA sequence and genome organization are similar to SPP1. Phages GBK2 and PM1 have modules of the genome that are evolutionarily linked to SPP1 but other modules of essential genes code proteins without detectable similarity to SPP1 gene products. This mosaic genome organization brings to debate if such type of relatedness [122] has enough taxonomic value to include GBK2 and PM1 in the same taxon as SPP1. Therefore, we limit at present the SPP1-like genus proposal to its close genetic neighborhood until more robust phylogenetic and biological evidence is obtained to expand the genus to other phages.

## Figures and Tables

**Table 1:** Revision of the SPP1 sequence. Changes in the GenBank X97918.2 sequence relative to the GenBank X97918.3 revised version are listed. The position coordinates presented are the ones found in the GenBank X97918.3 sequence. The ORF concerned, the nucleotide sequence change(s), and the effect(s) on the gene product amino acid sequence are displayed.

Position (nt)	ORF	ORF Sequence Change	ORF Product Amino Acid Change
230	*1*	insertion of a G; frameshift	gp1 Cter sequence changed and shortened
766	*2*	T→C	none (silent mutation)
1997	*4*	insertion of an A; frameshift	gp4 Cter changed and lengthened
5001	intergenic region ORFs *7* and *8*	insertion of a T	none
23,794	*30*	deletion of a C; frameshift	gp30 Cter changed and lengthened
34,450	*37.1*	T→G	gp37.1 C_18_→G_18_
37,304 and 37,306	*40*	GGC→CGG	gp40 R_194_R_195_→P_194_G_195_
38,371 to 38,374	*41*	GTGT→TGTG	gp41 K_101_C_102_→N_101_V_102_
40,240	*44* and former *45*	insertion of a G; frameshift; ORFs fusion	gp44 Cter lengthened by former gp45 sequence
41,787	*48*	insertion of an A; frameshift	gp48 changed and shortened
42,750	*51*	insertion of an A; frameshift	gp51 changed and lengthened
42,778 to 42,781	*51*	CGCG→GCGC	gp51 R_56_E_57_→A_56_Q_57_
42,819	*51*	insertion of a G; frameshift	gp51 Cter lengthened

**Table 2:** Rho-independent transcriptional terminators of SPP1. The terminators were identified as described in Material and Methods (see Section 2). Putative stem (underlined) and loop (bold) regions are highlighted. The coordinates of the sequence presented are shown (nt—nucleotide). The ΔG° of the RNA stem loop is shown on the right column.

**Terminator Sequence** **(Loop,** Stem **)**	**Start (nt)**	**End (nt)**	**ΔG° (kcal.mol^−1^)**
AAAAGCGAGTTAACCGACG**TAAAAATG**CGTCGGTTTTTTTCGTGCTTC	4718	4765	−12.20
GTGTCAGTGCGCGGGTTC**AATTCCCG**GAGTCCGTTTTTACCGCCC	6780	6824	−5.50
TAAAAAGAGAAGAGGGGC**TAAC**GCCTCTCTTTTTTTGAAAG	8775	8815	−14.00
ATTGCCAGCAGAGAGCAC**GGGTTAATTCC**CGCGCTCTTTTTTTGTATTCA	11,255	11,304	−9.20
TGAATTAGACAGGGCCGC**GCAA**GTGGCTCTTTTTAATAGGT	12,009	12,049	−15.20
TGAAAAGACGCGGCGCCG**CTAA**CGGCGTCCGTTTTGAACATGA	16,711	16,753	−12.10
GATTGACGAAGTAAAGGGC**CATGT**GCCCTTTATTTTTTTGCAAA	21,897	21,940	−10.70
ACCTTCGCTTGCCGCCCG**GCTGA**TGGGCGGTTTTTTATTTTT	24,095	24,136	−13.20
TGTCCTAAAATCGGCCCGT**TCCCAG**TCGGGCCACTTTTTTTATTTTA	27,934	27,980	−13.60
GACTTTGAAAGGAACCGTT**CTCT**AACGGTTCTTTTTTTATTTC	30,205	30,247	−9.90
TATTTTGATTGAGTCGGGG**AA**ACCCGGCTTTTTTATTTTGGG	35,232	35,273	−14.90
ATCAAAGTTATGGTGGGA**GTAA**TCCCGCCTTTTTCTATTTT	40,466	40,506	−14.40
AACACAGAGAGGCACCC**TATTT**GGGTGCTTTTTTGTTGTA	43,774	43,813	−11.90

**Table 3:** SPP1 ORFs. The 80 ORFs of the SPP1 transcribed heavy chain were assigned as described in Material and Methods (see Section 2) and numbered following the original nomenclature of Alonso et al. [26]. The RBS are sequences complementary to the *B. subtilis* 16 S rRNA 3′ sequence (mismatches are shown in small case; the position after which spacing is calculated between the RBS and the nucleotide preceding the initiation codon is underlined; n.d.—not determined). The ORFs coding regions coordinates are listed. Genes are defined essential when their inactivation in conditional lethal mutants prevents phage multiplication. The length, molecular mass (MM) and presence of putative transmembrane segments in proteins translated from the ORFs coding frame are listed on the right side of the Table. The coordinates of X-ray crystallography, NMR or cryo-electron microscopy structures available for individual proteins or their complexes are also provided. The function of individual proteins is based on experimental data while putative function is deduced from bioinformatics analysis. Structural components of the SPP1 viral particle (st.) are assigned based on biochemical, structural and/or robust bioinformatics data. Proteins are grouped according to function following the color code used in Figure 1.

ORF									Protein					
ORF	RBS (mRNA)	Spacing	Start	nt	Stop	nt	Essential		Length	MM	Predicted TMM	3D Structure	Protein Function	References
							Gene		(aa)	(kDa)	Segments	(PDB or EMD)		
***1***	AAGGAGGU	10	AUG	43,884	UGA	311	yes		147	16.3	no	3ZQQ ^a^ (Xtal ^b^)	small terminase subunit (TerS)	[57,63,64,65,66,67,68,69]
***2***	n.d. ^c^		AUG ^d^	308 ^d^	UAG	1576	yes		422 ^d^	48.8 ^d^	1 ^e^	2WBN ^f^; 2WC9 ^f^ (Xtal ^b^)	large terminase subunit (TerL)	[57,67,70,71,72,73,74]
***3***	AAAGGAGG	11	AUG	1567	UAA	1782	no		71	8.5	no	n.d.	unknown	
***4***	GGgGGU	10	AUG	1782	UAA	2072	no		96	11.4	no	n.d.	unknown	
***5***	AAGGAGG	11	AUG	2065	UGA	2334	no		89	10.3	no	n.d.	unknown	
***6***	AGGAGGU	11	AUG	2336	UGA	3847	yes		503	57.3	no	2JES (Xtal ^b^);	st.; portal protein	[20,60,67,73,74,75,76,77,78,79,80,81,82]
												5A20, 5A21 (cryoEM ^g^)		
***7***	AGGAGG	12	AUG	3804	UAA	4730	no		308	35.1	no	n.d.	st.; initiation of infection; binds to portal	[83,84]
***8***	AAAGGAG	12	AUG	5067	UGA	6215	n.d.		382	43.7	no	n.d.	unknown	
***9***	AAcGGAGG	9	AUG	6217	UAA	6555	n.d.		112	12.6	no	n.d.	unknown	
***10***	GGuGGUG ^h^	12 ^h^	AUG	6583	UAG	6750	n.d.		55	6.2	no	n.d.	unknown	
***11***	AGGAG	9	AUG	6917	UAA	7561	yes		214	23.4	no	n.d.	procapsid scaffolding protein	[85,86,87]
***12***	AAGGgGG	11	AUG	7576	UAA	7770	no		64	6.6	no	n.d.	st.; capsid accessory protein with collagen-like fold	[15,88]
***13***	AAAGGAG	9	AUG	7803	UAA	8777	yes		324	35.3	no	4AN5 (cryoEM ^g^)	st.; major capsid protein (MCP)	[15,85,86]
***14***	AAAGGAG	10	AUG	8828	UGA	9004	no		58	6.7	no	n.d.	unknown	
***15***	AAuGAGG	10	AUG	9015	UAA	9323	yes		102	11.6	no	2KBZ (NMR ^i^);	st.; connector adaptor protein	[60,78,81,89]
												5A20, 5A21 (cryoEM ^g^)		
***16***	n.d. ^c^		AUG ^j^	9325 ^j^	UAG	9654	yes		109 ^j^	12.5 ^j^	no	2KCA (NMR ^i^)	st.; connector stopper protein	[60,78,81,89]
												5A20, 5A21 (cryoEM ^g^)		
***16.1***	AAaGAGG	11	AUG	9644	UGA	10,069	n.d.		141	15.9	no	n.d.	putative tail protein	
***17***	AGGAGGU	10	AUG	10,066	UGA	10,470	yes		134	15	no	2LFP (NMR ^i^)	st.; tail-to-head joining protein (THJP)	[32,90]
***17.1***	AGGAGG	10	AUG	10,484 ^k^	UAA	11,017	yes		177	19.2	no	n.d.	st.; tail tube protein (TTP)	[16,91,92]
17.1*	AGGAGG	10	AUG	10,484 ^k^	UAA	11,279	no		264	28.2	no	n.d.	st.; tail tube protein; Cter FN3 motif	[91]
***17.5***	GAGG	12	AUG	11,363 ^l^	UAA	11,884	n.d. ^m^		173	20.2	no	n.d.	tail chaperone protein	[91]
17.5*	GAGG	12	AUG	11,363 ^l^	UAG	12,255	n.d. ^m^		297	34	no	n.d.	tail chaperone protein	[91]
***18***	AGGAGG	9	AUG	12,267	UGA	15,365	n.d. ^m^		1032	110.9	4 ^n^	n.d.	st.; tape measure protein (TMP)	[16]
***19.1***	GAGG	10	AUG	15,362	UAA	16,123	n.d. ^m^		253	28.6	no	2X8K (Xtal ^b^)	st.; distal tail protein (Dit)	[93,94]
***21***	AAGaAGGUGA	10	UUG	16,137	UAA	19,463	n.d. ^m^		1108	123.6	no	n.d.	st.; tail tip protein; Tal; anti-receptor protein	[93,95]
***22***	AAGGAGG	9	AUG	19,476	UAA	19,916	n.d.		146	16.7	no	2XC8 (Xtal ^b^)	putative tail protein	[96]
***23***	AGGAGGU	10	AUG	19,932	UGA	20,096	n.d.		54	6.1	no	n.d.	n.d.	
***23.1***	GGAG	9	AUG	20,089	UAA	20,244	n.d.		51	5.8	no	2XF7 (Xtal ^b^)	n.d.	[97]
***24***	GgGGUG ^h^	10 ^h^	AUG	20,237	UAG	20,467	n.d.		76	8.4	no	n.d.	n.d.	
***24.1***	AAAGGgGG	11	AUG	20,547	UAA	20,825	n.d.		92	10.6	1	n.d.	component of holin; cell lysis	[33,98]
24.1*	AGGAGGU	10	AUG	20,574	UAA	20,825	n.d.		83	9.5	1	n.d.	component of holin; cell lysis	[33]
***25***	AAGGAG	12	AUG	20,845	UAA	21,660	n.d.		271	29.9	no	n.d.	endolysin; cell lysis	[33,98]
***26***	AAAGGAG	8	AUG	21,662	UAA	21,910	n.d.		82	9.4	2	n.d.	component of holin; cell lysis	[33,98]
26*	AAAGGAG ^o^	14 ^o^	AUG	21,668	UAA	21,910	n.d.		80	9.1	2	n.d.	component of holin; cell lysis	[33]
***26.1***	AAGGgGG ^o^	10 ^o^	AUG	22,009	UAG	22,152	n.d.		47	5.8	no	n.d.	unknown	
***27***	AAGGAGG	12	UUG	22,277	UAA	22,831	n.d.		184	20.8	no	n.d.	unknown	
***28***	GGAGG	9	AUG	22,834	UGA	23,121	n.d.		95	10.8	no	n.d.	unknown	
***29***	AGGAGG	13	AUG	23,069	UGA	23,371	n.d.		100	12	no	n.d.	putative DNA binding protein	
***29.1***	AGGgGG	9	GUG	23,358	UGA	23,675	n.d.		105	12.3	no	n.d.	unknown	
***30***	AGGgGG	10	AUG	23,675	UAA	23,854	n.d.		59	7.2	1	n.d.	unknown	
***30.1***	AGGgGG	9	AUG	23,859	UGA	24,029	n.d.		56	6.4	no	n.d.	unknown	
***31***	AAcGGAGGU	12	AUG	24,209	UAA	24,493	n.d.		94	11	no	n.d.	unknown	
***31.1***	GAGG	12	AUG	24,589	UAA	24,951	n.d.		120	12.9	3	n.d.	unknown	
***31.2***	GgGGUG ^h^	10 ^h^	AUG	24,964	UGA	25,281	no ^p^		105	11.5	2	n.d.	unknown	
***32***	GGAGGUG	8	AUG	25,278	UAA	27,788	no ^p^		836	96.3	no	n.d.	putative ATP-binding protein	
***32.5***	GGAGGUG	11	UUG	28,039	UAA	28,209	n.d.		56	6.7	no	n.d.	unknown	
***33***	AAAaGgGGU	11	AUG	28,226	UAA	29,995	no		589	64.9	no	n.d.	putative bacteria surface binding protein	
***33.1***	AAcGGAGG	9	AUG	30,011	UAA	30,229	n.d.		72	8.4	no	n.d.	unknown	
***34***	AGGgGG	9	AUG	30,364	UAG	30,522	n.d.		52	6.3	no	n.d.	putative transcriptional repressor	
***34.1***	AGGAGG	9	AUG	30,534	UGA	31,469	no		311	35.9	no	n.d.	5′-3′ exonuclease	[13,99]
***34.2***	GGAGG	12	AUG	31,466	UAA	31,639	n.d.		57	6.7	no	n.d.	unknown	
***34.3***	AAGGAGG	11	AUG	31,641	UAA	31,895	n.d.		84	9.8	no	n.d.	unknown	
***34.4***	GGAGG	11	AUG	31,897	UAA	32,187	n.d.		96	11.1	no	n.d.	unknown	
***35***	GGAGGU	11	AUG	32,177	UAG	33,040	yes		287	32	no	n.d.	recT-like recombinase	[13,100]
***36***	AGGAGGgGA	10	AUG	33,033	UAA	33,512	no		159	17.1	no	n.d.	SSB	[101]
***36.1***	AAaGgGGUGA ^h^	10 ^h^	AUG	33,537	UGA	34,028	n.d.		163	18.9	no	n.d.	putative HNH endonuclease	
***37***	GGAGG	10	AUG	34,032	UGA	34,406	n.d.		124	14.3	no	n.d.	unknown	
***37.1***	GGAGG	11	AUG	34,399	UGA	34,992	n.d.		197	22.3	no	n.d.	putative poly-gamma-glutamate hydrolase	
***37.2***	GaAGG	14	AUG	34,989	UGA	35,243	n.d.		84	9.7	no	n.d.	unknown	
***37.3***	AGGAGG	11	AUG	35,400	UAG	35,573	n.d.		57	6.7	no	n.d.	putative DNA binding protein	
***38***	AAGGAGG	13	AUG	35,580	UGA	36,350	yes		256	30	no	n.d.	SPP1 origin binding protein	
													and replication re-start (PriA-like)	[56,102]
***39***	AGGAGG	9	AUG	36,347	UGA	36,727	yes		126	14.6	no	1NO1 (Xtal ^b^)	gp40 helicase loader	[56,103,104]
***40***	GAGG	11	AUG	36,724	UAA	38,052	yes		442	49.7	no	3BGW (Xtal ^b^)	replicative DNA helicase;	
													binds host DnaG and DnaX	[56,103,105,106,107,108,109]
***41***	AAAGGgGG	10	AUG	38,069	UGA	38,569	n.d.		166	19.1	no	n.d.	unknown	
***42***	AAAGGAG	11	AUG	38,566	UAA	38,961	no		131	16	no	n.d.	unknown	
***42.1***	GGAGG	9	AUG	38,964	UGA	39,134	no		56	6.5	no	n.d.	unknown	
***42.2***	GgGG ^c, q^	12 ^c, q^	AUG	39,131 ^q^	UAA	39,427	no		98 ^q^	10.7 ^q^	no	n.d.	unknown	
***43***	GGAGG	10	GUG	39,431	UGA	39,784	no		117	14.2	no	n.d.	unknown	
***44***	AAGGAG	11	AUG	39,777	UAA	40,487	no		236	27.5	no	n.d.	Holliday junction resolvase	[13,31]
***46***	GAGG	12	AUG	40,596	UAA	40,898	no		100	11.5	no	n.d.	unknown	
***46.1***	AGGAGG	9	AUG	40,898	UAA	41,209	no		103	11.7	3	n.d.	unknown	
***47***	AGGgGG	9	AUG	41,304	UGA	41,663	no		119	13.7	no	n.d.	unknown	
***48***	GGAG	13	AUG	41,645	UGA	41,995	no		116	13.2	no	n.d.	unknown	
***49***	GGAGG	9	GUG	42,075	UGA	42,248	no		57	6.5	no	n.d.	unknown	
***50***	AAGGAGG	9	GUG	42,245	UAA	42,418	no		57	6.8	no	n.d.	unknown	
***50.1***	AAAGGAGG	9	GUG	42,434	UGA	42,616	no		60	6.7	2	n.d.	unknown	
***51***	AAGGAGG	9	AUG	42,613	UAA	43,014	no		133	14.7	1	n.d.	unknown	
***51.1***	AAGGAG	9	AUG	43,027	UGA	43,182	no		51	6.1	1	n.d.	unknown	
***52***	AAAGGAG	10	AUG	43,179	UGA	43,421	no		80	9.5	no	n.d.	unknown	
***53***	AAAGGAG	10	AUG	43,405	UGA	43,611	no		68	7.4	1	n.d.	unknown	

^a^ structure of gp1 from the SPP1-related phage SF6 (83% amino acid sequence identity with SPP1 gp1); ^b^ X-ray crystallography structure PDB access code; ^c^ No RBS identified according to the criteria defined in Material and Methods (see Section 2). In case of ORF *42.2* a G-rich sequence is identified as a potential site for ribosome binding; ^d^ the gene *2* beginning and the resulting length of gp2 is based exclusively on the position of the initiation codon assigned during annotation as no RBS was identified (see Section 3.2 for details) ^e^ gp2 is not a membrane protein according to presently available biochemical data; ^f^ Structures of the gp2 nuclease domain (residues 232 to 422 of the gp2 amino acid sequence); ^g^ cryo-electron microscopy structure EMD access code; ^h^ The RBS sequence is compatible with different spacings relative to the ORF initiation codon. ^i^ NMR structure PDB access code; ^j^ the gene *16* beginning and the resulting length of gp16 is based on the position of the initiation codon assigned during annotation, as no RBS was identified, and on the amino terminus sequencing of gp16 (see Section 3.2 for details); ^k^ genes *17.1* and 17.1* have the same 5′ sequence because the product of 17.1* results from a +1 frameshift at the end of their common reading frame [91] (see Section 3.2 for details); ^l^ genes *17.5* and 17.5* have the same 5′ sequence as the product of 17.5* results from a putative −1 frameshift within their common reading frame [91] (see Section 3.2 for details); ^m^ the essential nature of SPP1 genes coding for the tail chaperones, TMP, Dit and Tal proteins was not demonstrated experimentally but their functional homologs are essential in the phage systems presently characterized; ^n^ the SPP1 TMP gp18 features four predicted transmembrane segments but it is an anticipated component of the phage particle occupying the tail tube internal space [16] (see Section 3.8 for details); ^o^ the same RBS is used for translation at the initiation codons of genes *26* and 26* [33] (see Section 3.6 for details); ^p^ the sequence inversion in SPP1*invmir* (Figure 1) disrupts ORF *31.2* and renders gene *32* promoterless indicating that they are non-essential; ^q^ poor RBS sequence which might not ensure putative ORF *42.2* translation.

**Table 4:** Codon usage bias in SPP1 and *B. subtilis*. The total number of codons (No) used in the complete set of ORFs of SPP1 (this work) or *B. subtilis* [47,48] and the fraction of each codon used to code a specific amino acid are listed. When the fraction value differs by more than 0.1 this variation of codon usage frequency is highlighted in bold. Rare codons (fraction of usage below 0.1) are underlined.

Amino Acid	Codon		Fraction	No	Amino Acid	Codon		Fraction	No	Amino Acid	Codon		Fraction	No
Ala	GCG	SPP1	0.26	268	Gly	GGA	SPP1	0.31	318	Pro	CCU	SPP1	0.22	107
		*B.s*	0.26	24,574			*B.s*	0.31	26,381			*B.s*	0.29	12,824
Ala	GCA	SPP1	0.27	272	Gly	GGU	SPP1	0.24	245	Pro	CCC	SPP1	0.08	42
		*B.s*	0.28	26,416			*B.s*	0.18	15,457			*B.s*	0.09	4001
Ala	GCU	SPP1	0.27	279	Gly	GGC	SPP1	0.25	262	Ser	AGU	SPP1	0.16	123
		*B.s*	0.25	23,062			*B.s*	0.34	28,493			*B.s*	0.11	8096
Ala	GCC	SPP1	0.20	201	**His**	**CAU**	**SPP1**	**0.53**	131	Ser	AGC	SPP1	0.22	170
		*B.s*	0.21	19,342			***B.s***	**0.67**	18,610			*B.s*	0.23	17,226
Arg	AGG	SPP1	0.18	130	**His**	**CAC**	**SPP1**	**0.47**	119	Ser	UCG	SPP1	0.10	79
		*B.s*	0.10	4788			***B.s***	**0.33**	9019			*B.s*	0.10	7717
Arg	AGA	SPP1	0.28	198	**Ile**	**AUA**	**SPP1**	**0.27**	261	Ser	UCA	SPP1	0.25	193
		*B.s*	0.26	13,077			***B.s***	**0.13**	11,517			*B.s*	0.24	18,053
Arg	CGG	SPP1	0.12	83	**Ile**	**AUU**	**SPP1**	**0.35**	335	Ser	UCU	SPP1	0.17	132
		*B. s*	0.15	7329			***B.s***	**0.50**	45,181			*B.s*	0.20	15,615
Arg	CGA	SPP1	0.10	74	Ile	AUC	SPP1	0.38	365	Ser	UCC	SPP1	0.11	83
		*B. s*	0.11	5115			*B.s*	0.37	32,872			*B.s*	0.13	9757
Arg	CGU	SPP1	0.18	125	Leu (s)	UUG	SPP1	0.24	246	Thr	ACG	SPP1	0.26	211
		*B. s*	0.18	8755			*B.s*	0.16	18,745			*B.s*	0.27	17,693
Arg	CGC	SPP1	0.14	99	Leu	UUA	SPP1	0.24	251	Thr	ACA	SPP1	0.47	379
		*B. s*	0.20	9444			*B.s*	0.20	23,338			*B.s*	0.41	27,117
**Asn**	**AAU**	**SPP1**	**0.45**	344	**Leu**	**CUG**	**SPP1**	**0.10**	107	Thr	ACU	SPP1	0.15	120
		***B.s***	**0.57**	27,137			***B.s***	**0.24**	28,295			*B.s*	0.16	10,620
**Asn**	**AAC**	**SPP1**	**0.55**	425	Leu	CUA	SPP1	0.13	139	Thr	ACC	SPP1	0.12	94
		***B.s***	**0.43**	20,861			*B.s*	0.05	6030			*B.s*	0.16	10,497
**Asp**	**GAU**	**SPP1**	**0.52**	442	Leu	CUU	SPP1	0.20	203	Trp	UGG	SPP1	1.00	190
		***B.s***	**0.64**	40,291			*B.s*	0.24	28,226			*B.s*	1.00	12,571
**Asp**	**GAC**	**SPP1**	**0.48**	415	Leu	CUC	SPP1	0.08	87	**Tyr**	**UAU**	**SPP1**	**0.50**	276
		***B.s***	**0.36**	22,699			*B.s*	0.11	13,232			***B.s***	**0.65**	27,650
Cys	UGU	SPP1	0.54	50	Lys	AAG	SPP1	0.38	460	**Tyr**	**UAC**	**SPP1**	**0.50**	278
		*B.s*	0.45	4429			*B.s*	0.30	25,647			***B.s***	**0.35**	14,673
Cys	UGC	SPP1	0.46	42	Lys	AAA	SPP1	0.62	760	Val (s)	GUG	SPP1	0.23	211
		*B.s*	0.55	5322			*B.s*	0.70	60,072			*B.s*	0.26	21,585
Gln	CAG	SPP1	0.38	192	Met (s)	AUG	SPP1	1.00	412	Val	GUA	SPP1	0.26	240
		*B.s*	0.46	22,750			*B.s*	1.00	32,918			*B.s*	0.20	16,296
Gln	CAA	SPP1	0.62	319	**Phe**	**UUU**	**SPP1**	**0.48**	270	Val	GUU	SPP1	0.32	292
		*B.s*	0.54	23,889			***B.s***	**0.68**	37,445			*B.s*	0.28	23,440
Glu	GAG	SPP1	0.39	452	**Phe**	**UUC**	**SPP1**	**0.52**	294	Val	GUC	SPP1	0.18	168
		*B.s*	0.32	28,211			***B.s***	**0.32**	17,253			*B.s*	0.26	21,143
Glu	GAA	SPP1	0.61	704	Pro	CCG	SPP1	0.44	219	**End**	**UGA**	**SPP1**	**0.39**	31
		*B.s*	0.68	59,808			*B.s*	0.43	19,421			***B.s***	**0.24**	965
Gly	GGG	SPP1	0.21	215	Pro	CCA	SPP1	0.26	127	End	UAG	SPP1	0.11	9
		*B.s*	0.16	13,670			*B.s*	0.19	8541			*B.s*	0.14	591
										**End**	**UAA**	**SPP1**	**0.50**	40
												***B.s***	**0.62**	2542
